# *DEFA1A3* DNA gene-dosage regulates the kidney innate immune response during upper urinary tract infection

**DOI:** 10.26508/lsa.202302462

**Published:** 2024-04-05

**Authors:** Jorge J Canas, Samuel W Arregui, Shaobo Zhang, Taylor Knox, Christi Calvert, Vijay Saxena, Andrew L Schwaderer, David S Hains

**Affiliations:** 1 Division of Pediatric Nephrology, Department of Pediatrics, Indiana University School of Medicine, Indianapolis, IN, USA; 2 Department of Microbiology and Immunology, Indiana University School of Medicine, Indianapolis, IN, USA; 3 Riley Hospital for Children, Indiana University Health, Indianapolis, IN, USA; 4 Kidney and Urology Translational Research Center, Herman B Wells Center for Pediatric Research, Indiana University School of Medicine, Indianapolis, IN, USA

## Abstract

Antimicrobial peptides (AMPs) are host defense effectors with potent neutralizing and immunomodulatory functions against invasive pathogens. The AMPs α-Defensin 1-3/*DEFA1A3* participate in innate immune responses and influence patient outcomes in various diseases. DNA copy-number variations in *DEFA1A3* have been associated with severity and outcomes in infectious diseases including urinary tract infections (UTIs). Specifically, children with lower DNA copy numbers were more susceptible to UTIs. The mechanism of action by which α-Defensin 1-3/*DEFA1A3* copy-number variations lead to UTI susceptibility remains to be explored. In this study, we use a previously characterized transgenic knock-in of the human *DEFA1A3* gene mouse to dissect α-Defensin 1-3 gene dose–dependent antimicrobial and immunomodulatory roles during uropathogenic *Escherichia coli* (UPEC) UTI. We elucidate the relationship between kidney neutrophil– and collecting duct intercalated cell–derived α-Defensin 1-3/*DEFA1A3* expression and UTI. We further describe cooperative effects between α-Defensin 1-3 and other AMPs that potentiate the neutralizing activity against UPEC. Cumulatively, we demonstrate that *DEFA1A3* directly protects against UPEC meanwhile impacting pro-inflammatory innate immune responses in a gene dosage–dependent manner.

## Abstract (precis)

α-Defensin 1-3 (*DEFA1A3*) are host antimicrobial peptides with potent innate immune functions during infectious diseases. Differential UTI risk has been linked to *DEFA1A3* DNA polymorphisms. This study elucidates mechanisms of *DEFA1A3* gene dose–dependent protection against UTI pathogenesis.

## Introduction

Urinary tract infections (UTIs) represent one of the most common infections in children (1). Although many anatomical and physiological genetic risk factors have been identified, our understanding of host immunological susceptibility to UTIs remains incomplete (2, 3). The innate immune system acts as the primary line of defense against infections orchestrating a rapid, complex host response (4, 5, 6). Recognition of conserved pathogenic cues such as bacterial cell wall, flagella, or nucleic acids enables the rapid signaling induction of cytokines, antimicrobial peptides (AMPs), and damage-associated molecular patterns (DAMPs) (7, 8). α-Defensin 1-3 are AMPs that participate in the innate immune response to exert both neutralizing and immunomodulatory roles against microbes. Previous studies have characterized α-Defensin 1-3 as a novel biomarker in pediatric and adult UTIs that can be expressed by neutrophils and collecting duct epithelia in the infected kidney (9, 10, 11, 12, 13, 14, 15). Encompassing 5–12% of the human genome, DNA copy-number variations (CNVs) are repeated genomic regions that can affect the expression of a gene in a dosage-dependent manner, and the *DEFA1A3* locus ranges between 2 and 18 diploid copies in individuals (16, 17). Previous studies have demonstrated that α-Defensin 1-3 levels increase in UTI patients, and low *DEFA1A3* DNA CNVs are associated with a higher risk of recurrent UTIs in children with vesicoureteral reflux (10, 18, 19). Because mice lack a homolog, investigating the role of *DEFA1A3* DNA CNVs in the mechanism(s) of host defense against uropathogens has not been extensively explored. To circumvent these limitations, a transgenic mouse with a knock-in of the human *DEFA1A3* gene has been used to study α-Defensin 1-3/*DEFA1A3* roles using in vivo models of UTIs (9, 20, 21).

There are multiple remaining questions regarding *DEFA1A3’s* role with innate immunity and UTI pathophysiology. The biological consequences of different *DEFA1A3* copy numbers with respect to gene dosage mechanisms have yet to be defined. Because α-Defensin 1-3/*DEFA1A3* is expressed in both leukocytes and collecting duct epithelial cells, the cooperativeness and differential involvement in the innate immune response to UTIs from these distinct cell types need to be further addressed. α-Defensin 1-3 and other AMPs expressed during UTI pathophysiology have been robustly characterized by their ability to disrupt bacterial membranes (12, 22, 23, 24, 25, 26). However, numerous studies have increasingly recognized a wide variety of immunomodulatory innate immune roles; thus, the involvement of *DEFA1A3* CNV differences across infectious immune responses warrants investigation (14, 15, 21, 27, 28, 29, 30). In addition, α-Defensin 1-3 at mucosal surfaces can be expressed in conjunction with other AMPs and innate immune components against microbes (31). Studies to further expand the understanding of synergistic effects could further provide translational utility against drug-resistant uropathogens (32, 33, 34, 35). The objective of this study was to evaluate the mechanistic role of α-Defensin 1-3/*DEFA1A3* protection in the kidney against uropathogenic *Escherichia coli* (UPEC) infection burden and inflammation.

## Results

### After UPEC superinfection challenge, *DEFA1A3* is expressed in gene dose–dependent fashion, and higher mRNA expression correlates with a lower bacterial burden

**Figure 1.**
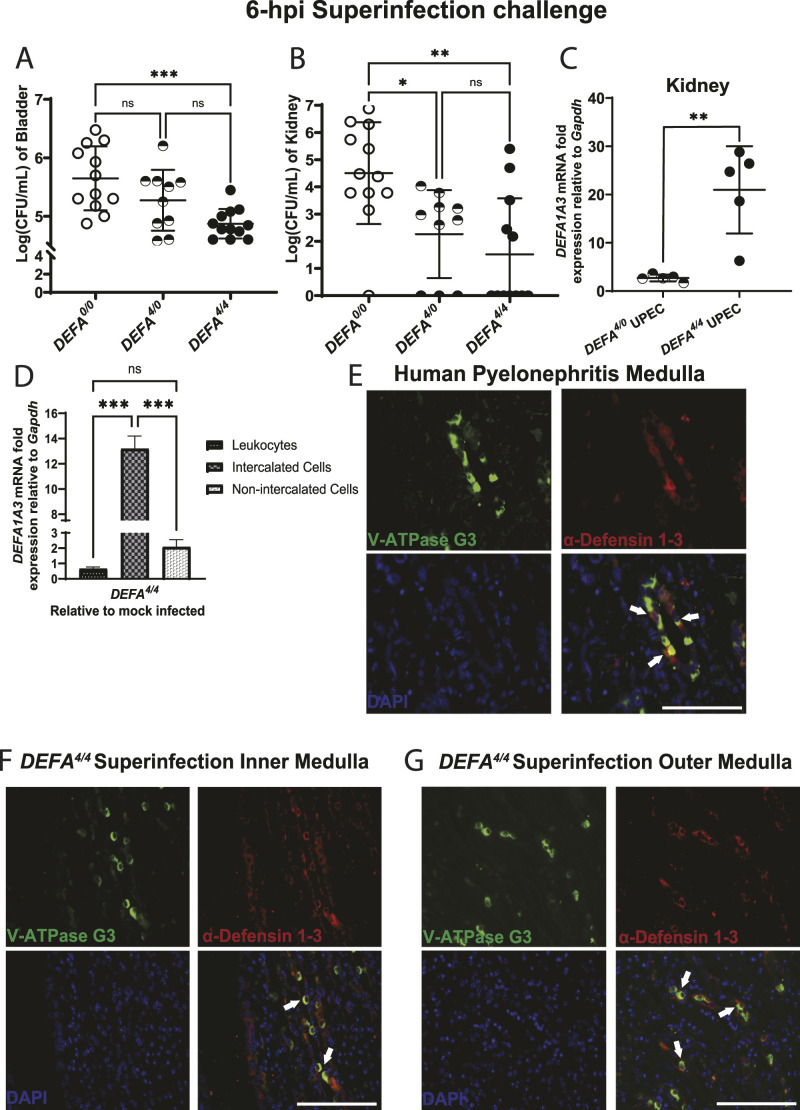
Human *DEFA1A3* gene transgenic mice are protected against urinary tract infections in a gene dose–dependent manner. **(A, B)** Quantified CFU/ml of (A) bladder and (B) pooled kidney lysates from infected mice with 0, 4, and 8 human *DEFA1A3* gene copies after 6 h of uropathogenic *Escherichia coli* (UPEC); CFT073 superinfection challenges. Statistically, comparisons were analyzed using one-way ANOVA with Tukey’s post hoc test from 12 biological replicates per group. **(C)** Infected kidneys from *DEFA*^*4/0*^ and *DEFA*^*4/4*^ mice were compared for inducible *DEFA1A3* mRNA expression levels. Data are represented as five individual biological replicates with the representation of the mean ± SD for each plot and compared with a *t* test. **(D)** Measurement of *DEFA1A3* mRNA expression was performed in magnetic-sorted kidney-derived leukocytes (CD45^+^), intercalated cells (CD45^−^CD117^+^), and non-intercalated cells (CD45^−^CD117^−^) from mock and UPEC-challenged *DEFA*^*4/4*^ mice normalized to housekeeping *Gapdh* expression. Bar graphs are representative of the mean ± SD from the fold ratio of UPEC over mock-infected mice displaying mRNA expression from six total biological replicates and compared via one-way ANOVA and Tukey’s post hoc test. **(E)** Evaluation of human pyelonephritis kidney medulla sections stained for V-ATPase G3 (green), α-Defensin 1-3 (red), and nuclear DAPI (blue) immunofluorescence markers. **(F, G)** UPEC-infected *DEFA*^*4/4*^ mouse kidney sections representative of (F) inner and (G) outer medullary kidney regions are imaged for similar markers. Intercalated cells (V-ATPase G3^+^) expressing α-Defensin 1-3 were identified with white arrows. The scale bar represents 100 μm size for the respective section recorded under a 60X objective lens.

We evaluated the α-Defensin 1-3/*DEFA1A3* gene dose–dependent mechanism(s) of protection by inducing UTIs with double transurethral inoculation of UPEC; CFT073, 3 h apart, as previously described in the superinfection challenge (36). In our previous study, kidney *DEFA1A3* mRNA expression inversely correlated with bacterial burden under single and superinfection UPEC challenges (9). Because both PMNs and collecting duct epithelial cells can express α-Defensin 1-3, the inducible cellular source of *DEFA1A3* expression that inversely correlates with the lower bacterial burden phenotype remains to be dissected. Previous studies have characterized an increased period of bacteriuria, infection of urinary tract organs, and acute inflammation after the UPEC superinfection challenges under the C57BL/6J background (36). Comparing the superinfection-challenged mice with absent, low, and high human *DEFA1A3* DNA copy numbers (*DEFA*^*0/0*^, *DEFA*^*4/0*^, and *DEFA*^*4/4*^), our results indicate transgenic *DEFA*^*4/4*^ mouse bladders displayed significantly lower average mean bacterial CFUs at 6 hpi compared with *DEFA*^*4/0*^ and *DEFA*^*0/0*^ mice (Fig 1A). Kidney tissues from *DEFA*^*4/0*^ and *DEFA*^*4/4*^ mice showed a decrease in CFU burden compared with *DEFA*^*0/0*^ (Fig 1B). After superinfection challenges at 6 hpi, *DEFA*^*4/4*^ (8 total copies) kidneys significantly increased *DEFA1A3* mRNA expression by an average of 20-fold, far exceeding the twofold increase in infected *DEFA*^*4/0*^ (four total copies) mouse kidneys (Fig 1C). Collectively, these results suggest transgenic *DEFA1A3* DNA copy number–dependent mRNA expression is inversely proportional to bacterial burdens in the kidney and bladder.

### Induction of intercalated cell α-Defensin 1-3/*DEFA1A3* expression contributes to reduced urinary tract bacterial burden under the UPEC superinfection challenge

**Figure S1.**
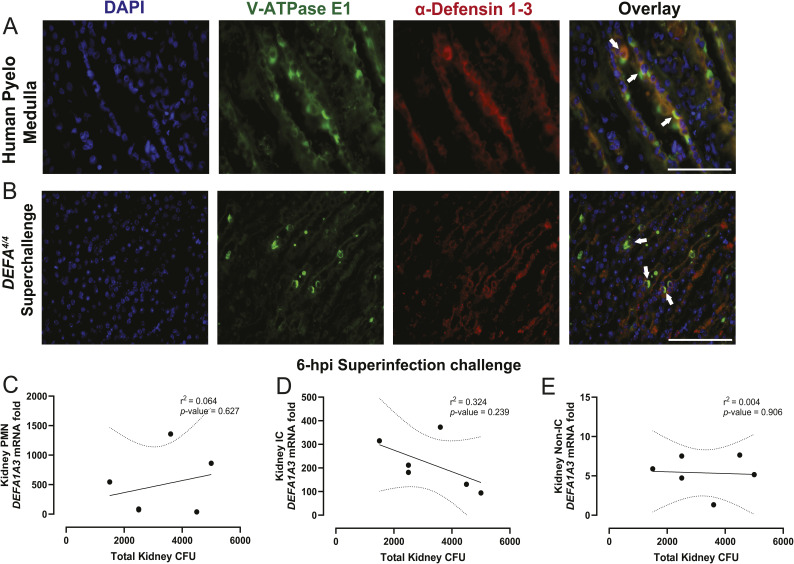
Human and mouse medullary collecting duct–derived intercalated cells co-localize α-Defensin 1-3 expression. **(A, B)** Pyelonephritis human– and (B) uropathogenic *E. coli*–challenged *DEFA*^*4/4*^ mouse kidney sections were stained with V-ATPase E1 (green), α-Defensin 1-3 (red), and nuclear DAPI (blue) markers. Arrows denote co-location events from α-Defensin 1-3–expressing collecting duct epithelial cells (suspected intercalated cells). The white bar indicates 100 μm size for the image sections recorded under a 60X objective lens. **(C, D, E)** Correlation analysis with indicative Pearson’s coefficients and *P*-values from uropathogenic *E. coli*–challenged total kidney bacterial CFUs from isolated (C) leukocytes, (D) intercalated cells, and (E) non-intercalated cells at 6 hpi. The straight line denotes the linear regression with 95 confidence intervals represented with dotted lines for correlation graphs.

Because transcription and translation of PMN-derived α-Defensin 1-3/*DEFA1A3* occur during promyelocytic stages in the bone marrow, we evaluated the possibility that α-Defensin 1-3 from other cellular sources represent the increased *DEFA1A3* mRNA expression in the infected kidney (37, 38). Collecting duct epithelial–derived ICs were postulated as the source of *DEFA1A3* induction in the kidney because of shared physiology with other constitutive and inducible renal AMPs (22, 39, 40). After enrichment of kidney-derived PMNs, collecting duct epithelial ICs, and kidney cells that are not intercalated cells (non-ICs), we performed quantification of *DEFA1A3* gene expression that reveals the increased mRNA expression of 13.2-fold ± 0.71 in ICs after the UPEC superinfection challenge compared with other cell types (Fig 1D). Our results indicate an inverse relationship between kidney bacterial CFUs and IC-*DEFA1A3* mRNA expression upon induction (*r* = −0.570), but not in *DEFA1A3*-expressing PMN sources (*r* = 0.254) (Fig S1C). Non-IC–derived *DEFA1A3* mRNA expression lacked correlation relationships to bacterial CFUs in the kidney (*r* = −0.063), although non-IC mRNA expression increased to a lesser extent than ICs (2.1 ± 0.33 mRNA fold expression) (Figs 1D and S1D and E). To confirm expression at the protein level, we analyzed immunofluorescence co-localization of α-Defensin 1-3 with structural kidney cells by staining human pyelonephritis, and mouse post-challenged kidney sections with the IC marker (V-ATPase G3) (Fig 1E–G). We confirmed α-Defensin 1-3 co-localization with V-ATPase E1 marker for ICs in both human and mouse kidney sections (Fig S1A and B). The results suggest collecting duct epithelial intercalated cells primarily induce α-Defensin 1-3/*DEFA1A3* expression to a lower bacterial burden in the setting of UPEC-induced superinfection.

### PMNs, but not PMN-derived α-Defensin 1-3/*DEFA1A3* expression, are critical to urinary tract defense after the UPEC superinfection challenge

**Figure 2.**
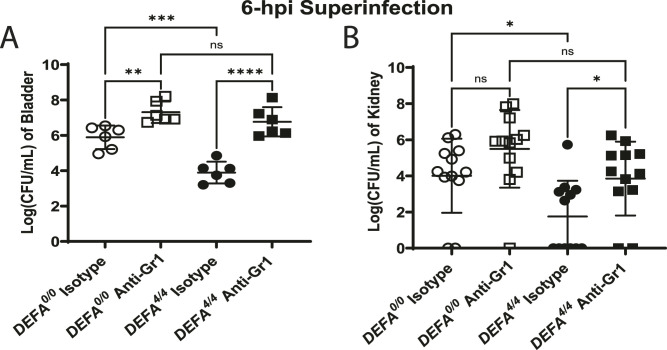
Neutrophils coordinate *DEFA1A3*-mediated urinary tract infection burden protection against uropathogenic *E. coli* in vivo. **(A, B)** At 6 hpi of CFT073 superinfection challenge, (A) bladder and (B) individual kidney lysates from anti-Gr1– and isotype-treated *DEFA*^*0/0*^ and *DEFA*^*4/4*^ littermate mice were quantified for bacterial burdens and are represented as the mean CFU/ml ± SD of the respective organ from six biological replicates. One-way ANOVA with Tukey’s post hoc test was applied to evaluate differences between groups.

Upon uropathogen invasion, PMNs migrate from the bloodstream to the kidney to release antimicrobial effectors stored in granules (41). To determine whether neutrophils are necessary to mediate *DEFA1A3*-dependent antimicrobial effects, we depleted Gr-1^+^ cells in *DEFA*^*4/4*^ and *DEFA*^*0/0*^ littermate mice before superinfection challenges. After depletion of neutrophils and challenge, CFU analysis shows infected urinary tract tissues between mouse groups were not different in the bladder nor kidney tissues (Fig 2A and B). Urinary tract tissues from challenged *DEFA*^*4/4*^ mice with Gr1+ cell depletion had increased bladder and kidney bacterial burdens but to a similar degree as the WT counterpart (*P* = 0.528 for bladder and *P* = 0.291 for kidneys). In the infected urinary tract tissues of *DEFA*^*4/4*^ and mice after UTI challenges, Gr1^+^ cell depletion led to increased bacterial burdens compared with IgG isotype treatment (Fig 2). Thus, neutrophils are needed to confer urinary tract protective phenotypes against UPEC independent of their α-Defensin 1-3 production. Interestingly, the *DEFA*^*4/4*^ mouse kidneys had lower bacterial burdens in the kidneys compared with WT after Gr1^+^ cell depletion, indicating some protective effect potentially from renal α-Defensin 1-3 production similar to our transplant mouse data in previous work (Fig 2B) (9).

### α-Defensin 1-3 permeabilize bacterial membranes and agglutinate *E. coli* strains in time- and dose-dependent manners

**Figure 3.**
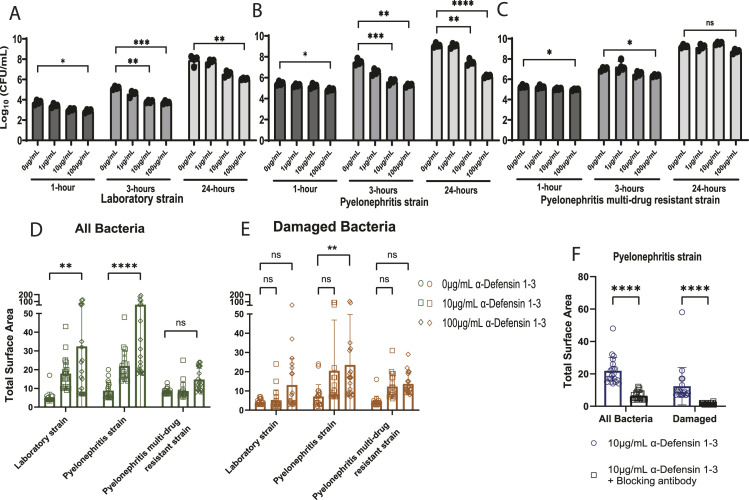
α-Defensin 1-3 induce dose-dependent bacterial membrane damage and cell agglutination of *E. coli* strains. **(A, B, C)** Time-kill quantification of bacterial growth at increasing concentrations (1, 10, and 100 μg/ml) and co-incubation times (1, 3, and 24 h) for (A) laboratory *E. coli* strain; K12, (B) pyelonephritis strain; CFT073, and (C) pyelonephritis multi-drug–resistant uropathogenic *E. coli* strain; MDR58. The mean CFU/ml ± SD for duplicate technical replicates from two separate experiments is reflected in bar graphs. Bacterial co-incubations after 3 h at 37°C of co-incubation with the respective *E. coli* strain and various α-Defensin 1-3 peptide concentrations. **(D, E)** Total surface area of bacterial aggregates (μm^2^) quantified in (D) SYTO9 staining images to identify all bacteria and (E) PI staining images of damaged and dying *E. coli* aggregates. **(F)** Similarly, quantification of bacterial agglutination of all bacteria (left) and damaged bacteria (right) after co-incubation with (F) anti-α-Defensin 1-3 antibody and α-Defensin 1-3 peptides with pyelonephritis strain; CFT073. Two-way ANOVA with Sidak’s multiple comparison test was applied to analyze conditions.

**Figure S2.**
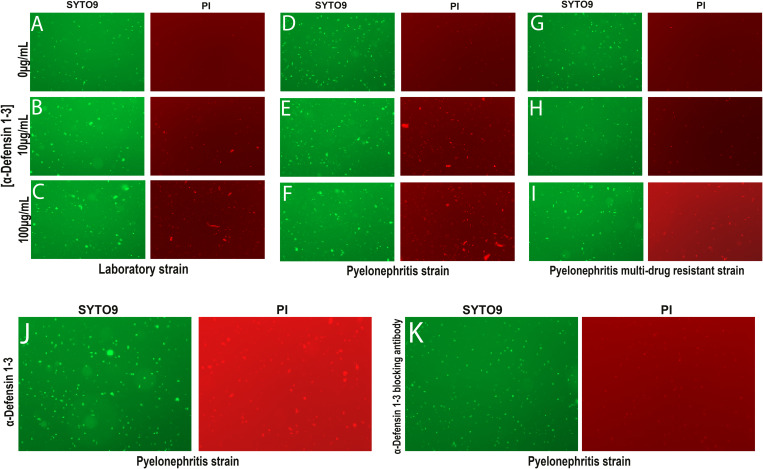
α-Defensin 1-3 exert bacterial agglutination alone and in combination with other antimicrobial peptides against *E. coli* strains. At 3 h after co-incubations, SYTO9 (green, left) and propidium iodide (red, right) staining images were recorded under a 20X objective fluorescent lens. **(A, B, C, D, E, F)** Laboratory *E. coli* strain; K12 and (D, E, F) pyelonephritis strain uropathogenic *E. coli*; CFT073 displayed a time- and dose-dependent increase in agglutination of both all and membrane-damaged bacteria. **(G, H, I)** MDR58 demonstrated diminished bacterial damage and agglutination compared with other *E. coli* strains. **(J)** Visible agglutination (SYTO9) and membrane damage (PI) induced by 3 h of co-incubation with 10 μg/ml α-Defensin 1-3. **(K)** These effects were nulled when anti-α-Defensin 1-3 blocking antibody was added to the co-incubation. Images from staining were recorded under a 20X fluorescent microscope lens.

α-Defensin 1-3 agglutinate *Escherichia coli* in addition to permeabilizing and blebbing bacterial membranes (26, 32, 42). To test whether these effects were dependent on time exposure and dosage, we established time-kill antimicrobial assays that co-incubate α-Defensin 1-3 peptides with three increasing sub-inhibitory concentrations against laboratory; K12 and pyelonephritis; CFT073 *E. coli* strains (Fig 3A and B). α-Defensin 1-3 at a minimum of 10 μg/ml concentration significantly decreased bacterial counts as early as 3 and 24 h post-incubation after being sub-cultured overnight for CFU measurements in both strains. α-Defensin 1-3 had minimal bacteriostatic and bactericidal capacity against pyelonephritis multi-drug–resistant strain; MDR58 at 3 h, and viable bacterial counts recovered to baseline after 24 h post-incubation (Fig 3C). We used fluorescent dyes to visualize and quantify direct antimicrobial effects and agglutination. Propidium iodide (PI) identifies damaged/permeabilized bacteria, and SYTO9 stains all bacteria (Fig S2); hence, these can be used to see live versus dead bacteria by comparing SYTO9 with PI. At 3 h post-incubation, the in vitro assays indicated bacterial membrane permeabilization and agglutination at 10 μg/ml of α-Defensin 1-3 compared with vehicle-treated *E. coli* strains (Fig S2A–F). When the concentration was increased to 100 μg/ml, α-Defensin 1-3 peptides were able to induce higher significant agglutination effects similarly visualized across *E. coli* strains as evidenced by immunofluorescence (Fig S2C and F). Furthermore, MDR58 co-incubation with α-Defensin 1-3 at 10 μg/ml lacked membrane permeabilization and agglutination effects after 3 h (Fig S2G and H).

We then quantified the total surface area of agglutination in all bacteria (SYTO9) and damaged (PI) bacterial images across *E. coli* laboratory and pyelonephritis strains with escalating concentrations of α-Defensin 1-3 (Fig 3D and E). α-Defensin 1-3 concentrations led to a dose-dependent positively correlated increase in bacterial agglutination with significantly larger “clumps” of live and damaged bacteria in the laboratory strain and pyelonephritis strain; CFT073. Significant agglutination was quantified for both channels using the 100 μg/ml concentration of α-Defensin 1-3 peptides (*P* = <0.0001 and 0.006, respectively) compared with vehicle co-incubations in the pyelonephritis strain. Co-incubations of multi-drug–resistant UPEC; MDR58 and α-Defensin 1-3 peptides did not result in significant bacterial agglutination (*P* = 0.999 and 0.705) in neither of recorded SYTO9 nor PI at 100 μg/ml of α-Defensin 1-3 peptides (Fig S2I). The agglutination and antimicrobial activity were eliminated after co-incubation with anti-α-Defensin 1-3 blocking antibody (Figs 3F and S2J and K). Cumulatively, α-Defensin 1-3 direct membrane permeability and agglutination are bactericidal effects that occur in a time- and dose-dependent manner against *E. coli* and are dependent on pathogen-specific factors such as virulence or antibiotic resistance mechanisms.

### α-Defensin 1-3 have complementary antimicrobial effects with LL-37, RNase7, and DMBT1 peptides against multi-drug–resistant UPEC

**Figure S3.**
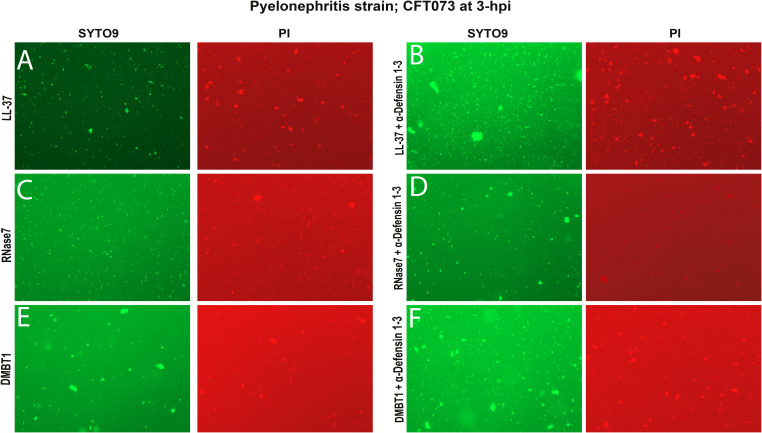
α-Defensin 1-3 peptide bacterial damage and agglutination are enhanced in combination with LL-37, RNase7, and DMBT1 antimicrobial peptides against pyelonephritis *E. coli* strain in vitro. **(A, B, C, D, E, F)** Visual agglutination of all bacteria (left) and damaged (right) pyelonephritis strain; CFT073 co-incubated with 30 μg/ml (A) LL-37, (C) RNase7, and (E) DMBT1 antimicrobial peptides alone or in combination with (B, D, F) 10 μg/ml α-Defensin 1-3 peptides. Images from staining were recorded under a 20X fluorescent lens.

**Figure 4.**
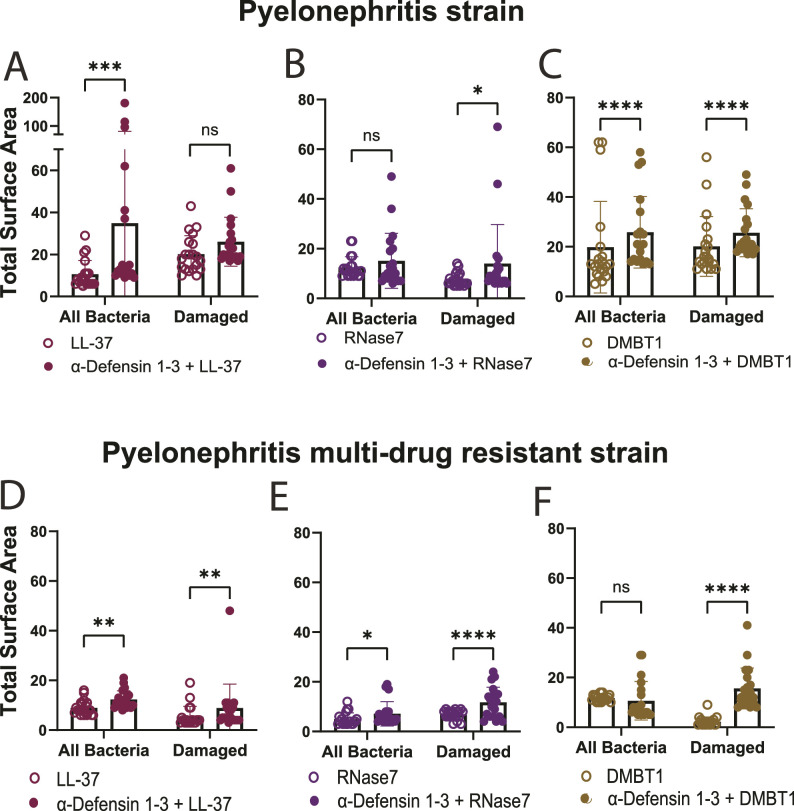
Combination of α-Defensin 1-3 peptides with LL-37, RNase7, and DMBT1 antimicrobial peptides potentiates bacterial agglutination against pyelonephritis and pyelonephritis multi-drug–resistant *E. coli* strains in vitro. After 3 h of co-incubation, alone, 30 μg/ml of LL-37, RNase7, and DMBT1 peptide co-incubations with pyelonephritis strain; CFT073 was imaged and quantified for bacterial agglutination. **(A, B, C)** Cooperative effects of both all bacterial and damaged bacterial agglutination representative quantification of 10 μg/ml α-Defensin 1-3 co-incubated with 30 μg/ml of (A) LL-37, (B) RNase7, and (C) DMBT1 peptides. **(A)** LL-37 manifested the largest recorded bacterial agglutination of pyelonephritis strain in combination with α-Defensin 1-3. Quantified bacterial agglutination of pyelonephritis multi-drug–resistant strain; MDR58 co-incubated with α-Defensin 1-3 peptides compared with LL-37, RNase7, and DMBT1 antimicrobial peptides at the same concentrations listed above. **(D, E, F)** Pyelonephritis multi-drug–resistant strain co-incubations with (D) LL-37, (E) RNase7, and (F) DMBT1 alone or in combination with α-Defensin 1-3 peptides. Data are represented as the mean ± SD of quantified total surface area of bacterial agglutination events recorded in μm^2^ scale. Two-way ANOVA and Sidak’s test were performed to compare various conditions.

**Figure S4.**
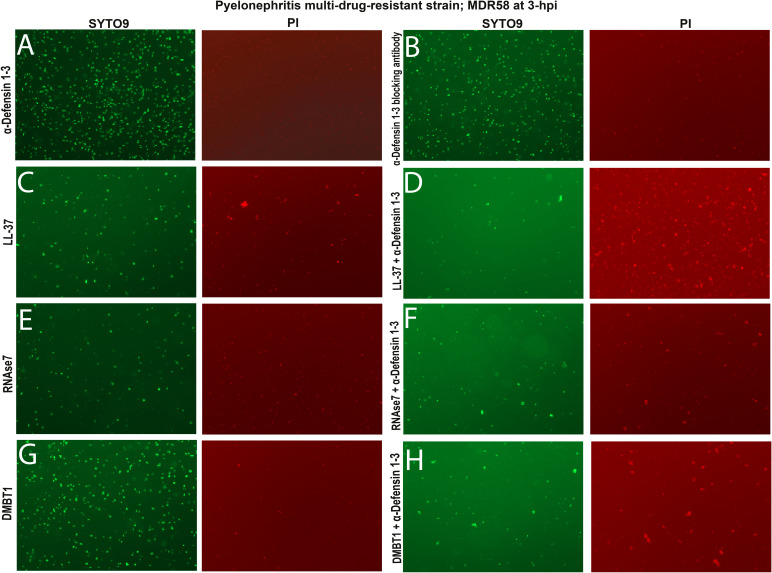
α-Defensin 1-3 peptide bacterial damage and agglutination are enhanced in combination with LL-37, RNase7, and DMBT1 antimicrobial peptides (AMPs) against pyelonephritis multi-drug–resistant *E. coli* strain in vitro. **(A, B, C, E, G)** Visual agglutination of all bacteria (left) and damaged (right) pyelonephritis multi-drug–resistant strain; MDR58 co-incubated with (A) 10 μg/ml α-Defensin 1-3, (B) anti-α-Defensin 1-3 blocking antibody, or 30 μg/ml of each AMP alone, (C) LL-37, (E) RNase7, and (G) DMBT1 peptides did not induce bacterial damaging effects as lacking PI staining. **(D, F, H)** Antimicrobial effects were visually enhanced when 10 μg/ml α-Defensin 1-3 was co-incubated with each AMP combination. **(D)** Combination of (D) α-Defensin 1-3 with either LL-37, RNase7, or DMBT1 peptides produced higher density of damaged bacterial aggregates at 3 h post-incubation than when AMP used alone. Images from staining were recorded under a 20X fluorescent lens.

Prior studies have initially elucidated how different AMPs orchestrate damaging responses against bacteria (9, 32, 34). We evaluated the ability of α-Defensin 1-3 to induce direct bacterial membrane permeability and agglutination effects in combination with known human AMPs LL-37, RNase7, and DMBT1. Prior research efforts have elucidated the antimicrobial activities of these AMPs against bacterial species including *E. coli* (9, 43, 44, 45). Alone, LL-37 and DMBT1 peptides induced membrane damage and agglutination after 3 h of co-incubation with pyelonephritis strain; CFT073 (Fig S3A and E). Alternatively, RNase7 only induced membrane damage but lacked agglutinating activity against bacteria (Fig S3C). By comparing SYTO9 and PI quantification of mean total surface area after co-incubations of different AMPs, we were able to assess agglutination versus bactericidal activity in a cooperative fashion against the pyelonephritis strain; CFT073 (Figs 4A–C and S3D). When co-incubated with α-Defensin 1-3, cooperative effects were observed with LL-37, DMBT1, and RNase7 based on bacterial agglutination (Fig S3B, D, and F). Quantification of agglutination indicates a significant increase in the mean total surface area of all bacteria when used in combination with LL-37 and DMBT1 peptides (*P* = 0.0008 and <0.0001) (Fig 4A and C). When compared to the PI staining, agglutination of viable bacteria increased with the combination of α-Defensin 1-3 and LL-37 (Fig 4A). A mild increase in the mean total surface area of damaged agglutinated bacteria for RNase7 and α-Defensin 1-3 mixtures was observed compared with the RNase7 co-incubations (*P* = 0.0318) (Fig 4B). The most significant effect on damaged bacterial agglutination was recorded with α-Defensin 1-3 in combination with DMBT1 (Fig 4C). We proceeded to evaluate the activity of these AMP combinations against pyelonephritis multi-drug–resistant strain; MDR58, using respective fluorescent dyes (Fig S4A–H). Although less mean bacterial agglutination total surface area was recorded, significant cooperative effects were quantified and compared with co-incubation of the candidate AMP mixtures against pyelonephritis multi-drug–resistant strain (Fig 4D–F). The most significant difference between pyelonephritis and MDR strains is the blunting of LL-37 and α-Defensin 1-3 combinatorial agglutination, whereas DMBT1 agglutination with killing seems rescued with the addition of α-Defensin 1-3 (Fig 4D and F). Our results indicate α-Defensin 1-3 work in concert with other AMPs to neutralize *E. coli* strains in relation to strain-specific factors.

### *DEFA1A3* gene dosage associates with reduced pro-inflammatory immune cell recruitment after UPEC superinfection

**Figure 5.**
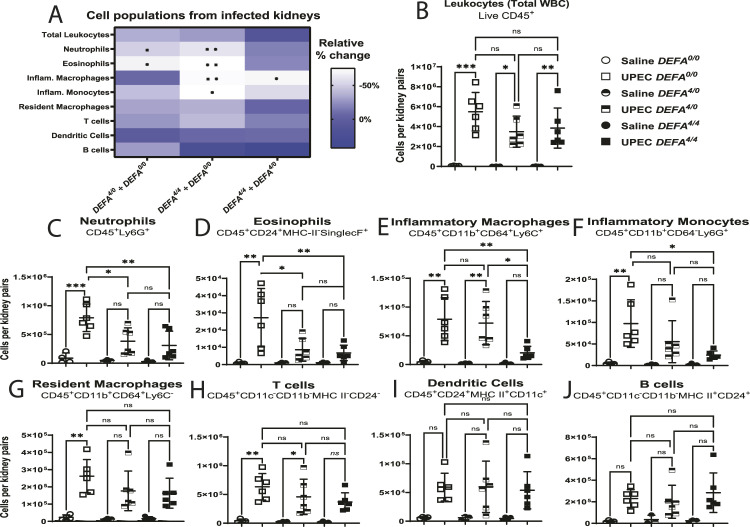
Human *DEFA1A3* gene transgenic mouse copies determine differential recruitment of pro-inflammatory cell populations after uropathogenic *E. coli* infection. **(A)** Relative percent change of immune cell populations was quantified to compare the responses between littermate *DEFA*^*0/0*^, *DEFA*^*4/0*^, and *DEFA*^*4/4*^ mice after 6 hpi. Significant differences are summarized with asterisk(s) for the respective genotype and cell population comparison. Scale denotes the relative percent (%) change of infected murine groups. **(B, C, D, E, F, G, H, I, J)** Total live leukocytes, (C) neutrophils, (D) eosinophils, (E) inflammatory macrophages, (F) inflammatory monocytes, (G) resident macrophages, (H) T lymphocytes, (I) dendritic cells, and (J) B lymphocytes from infected kidneys were counted and compared against each *DEFA* genotype. Asterisk(s) within each genotype comparison represent(s) significant statistical differences from one-way ANOVA and applied Tukey’s post hoc test for three to six biological replicates in the cell population assessed. No differences across immune populations were recorded in vehicle-challenged mice with 0, 4, and 8 *DEFA1A3* copies. **(E)** Pro-inflammatory macrophages were only significantly impacted by comparing infected *DEFA*^*4/4*^ with *DEFA*^*4/0*^ kidneys. Data are represented as the mean ± SD of cell numbers gated from pooled kidney using expression markers according to labels in individual cell populations.

Previous studies have characterized the effects of the human *DEFA1A3* gene in the resident immune cell composition of the transgenic mouse urinary tract at baseline (9). We evaluated a comprehensive flow cytometry panel of leukocyte immune cell surface markers in the kidneys of the infected *DEFA*^*4/4*^, *DEFA*^*4/0*^, and *DEFA*^*0/0*^ mice (Fig 5A–J). After UPEC superinfection challenges, we found pro-inflammatory immune cell populations to be mostly differentially recruited to the kidney between *DEFA1A3*-carrier and non-carrier mice (Fig 5A). The significant differences corresponded to the less immune influx of neutrophils (*P* = 0.004), eosinophils (*P* = 0.008), inflammatory macrophages (*P* = 0.007), and inflammatory monocytes (*P* = 0.015) comparing infected *DEFA*^*4/4*^ and *DEFA*^*0/0*^ kidneys (Fig 5C–F). Comparing *DEFA*^*4/4*^ and *DEFA*^*4/0*^ mice, the inflammatory macrophages were differentially recruited (*P* = 0.019) to the infected kidneys (Fig 5E). Our results suggest that high α-Defensin 1-3/*DEFA1A3* copy number dampens pro-inflammatory macrophage recruitment during the cellular immune recruitment response to UPEC.

### *DEFA1A3* DNA gene dose–dependent antimicrobial effects lead to down-regulation of Toll-like receptor expression in infected kidneys

**Figure S5.**
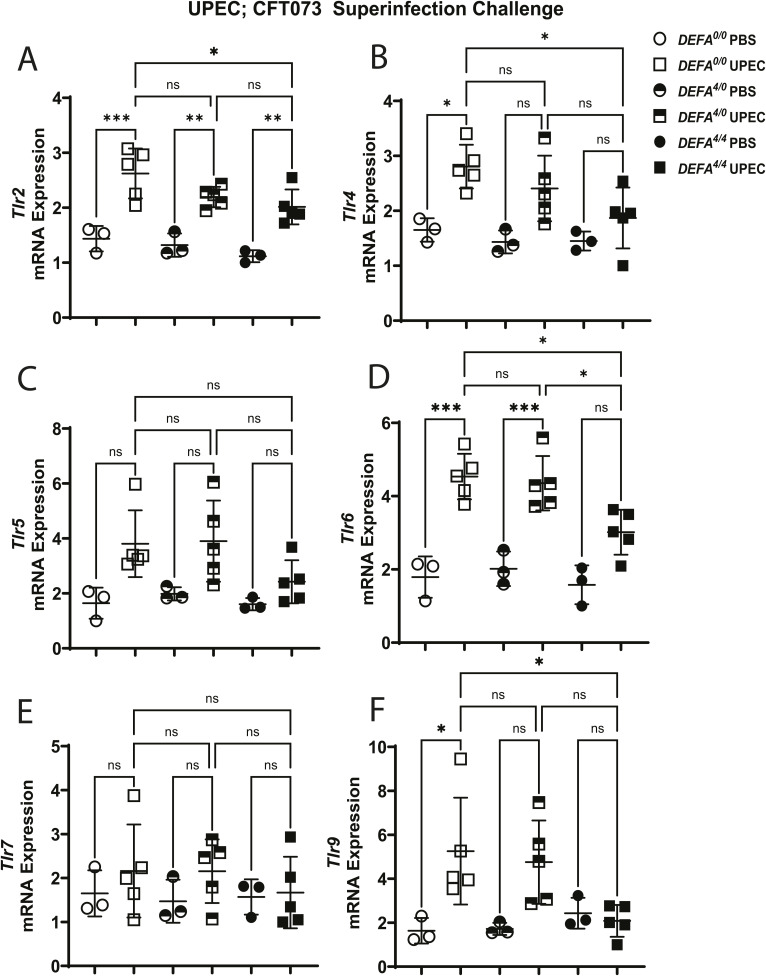
Human transgenic *DEFA1A3* copy numbers associate with reduced Toll-like receptor induction after uropathogenic *E. coli*; CFT073 superinfection. Screening of Toll-like receptor superfamily was performed via RT–qPCR from *DEFA1A3*-carrier and non-carrier infected kidneys with 0, 4, and 8 human gene copies. **(A, B, D, F)** After uropathogenic *E. coli* superinfection, mRNA expression differences for (A) *Tlr2*, (B) *Tlr4*, (D) *Tlr6*, and (F) *Tlr9* gene targets at 6 hpi between *DEFA*^*0/0*^ and *DEFA*^*4/4*^ mice are represented as the mean ± SD. **(C, E)** No significant differences for (C) *Tlr5* and (E) *Tlr7* expression across groups. Statistical differences between groups with three to five biological replicates were measured using one-way ANOVA with Tukey’s post hoc test.

We then explored Toll-like receptor signaling as the possible innate immune mechanistic cue(s) responsible for differential pro-inflammatory responses against UPEC in *DEFA1A3*-expressing mice (6, 46). Extensive research has suggested α-Defensin 1-3 can inhibit bacterial cell wall synthesis and SOS repair mechanisms via interactions with different bacterial components and Toll-like receptor ligands such as cell wall precursor lipid II, RNAs, and RecA-ssDNA nucleoprotein interactions in vitro (26, 32, 47). To investigate whether these interactions confer a differential immune pathogen recognition response in vivo, we evaluated the induction of Toll-like receptor expression for *E. coli* cellular bacterial ligands under the UPEC superinfection model. Upon assessment of acute Toll-like receptor genes induced at 6-hpi CFT073 superinfection challenge, we demonstrate a differential significant up-regulation of *Tlr2*, *Tlr4*, *Tlr6*, and *Tlr9* genes at the transcription level after challenges between *DEFA1A3* copy number–carrier and non-carrier mice (Fig S5A–F). Although the *DEFA*^*4/0*^ and *DEFA*^*0/0*^ mice similarly increased *Tlr2*, *Tlr4*, and *Tlr6* mRNA gene expression (*P* = 0.252, 0.713, and 0.997, respectively), the *DEFA*^*4/4*^ mice show the diminished inducible expression of these Toll-like receptor genes (*P* = 0.044, 0.036, and 0.012, respectively). Similarly, *Tlr9* mRNA induction was elevated in the *DEFA*^*0/0*^ and *DEFA*^*4/0*^ mice to a comparable extent (*P* = 0.995). However, infected *DEFA*^*4/4*^ kidneys lacked significant induction of *Tlr9* mRNA expression levels compared with *DEFA*^*0/0*^ and *DEFA*^*4/0*^ mice (*P* = 0.04 and 0.05, respectively). On the contrary, inducible *Tlr7* mRNA expression trends were similar across challenged mouse groups. Overall, the results suggest high *DEFA1A3* DNA copy numbers drive selective blunting of Toll-like receptor mRNA expression after UPEC invasion in the kidney.

### High *DEFA1A3* DNA gene dosage kidney anti-inflammatory effects are independent of UPEC antimicrobial activity

**Figure 6.**
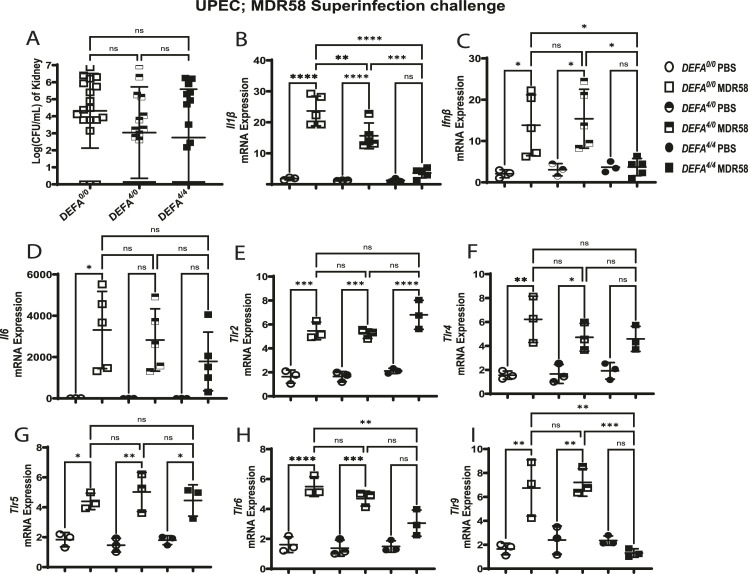
Human transgenic *DEFA1A3* DNA copies modulate kidney pro-inflammatory responses in a bacterial viability–independent manner. **(A)** Individual kidneys from *DEFA*^*0/0*^, *DEFA*^*4/0*^, and *DEFA*^*4/4*^ mice were quantified for CFU/ml after superinfection challenges with pyelonephritis multi-drug–resistant strain; MDR58 or vehicle. RT–qPCR from infected mouse kidneys was applied to assess induction of Toll-like superfamily and pro-inflammatory downstream gene targets. **(B)** Down-regulation of kidney *IL1β* mRNA gene expression follows a gene dose–dependent pattern in infected kidneys. **(C)** On the contrary, only *DEFA*^*4/4*^ mice displayed lower *IFNβ* mRNA expression levels compared with infected kidneys from littermates with 0 and 4 *DEFA1A3* copies. **(D)** *IL6* gene expression was non-significantly lower in infected *DEFA*^*4/4*^ kidneys. **(E, F, G, H)** Toll-like receptor targets quantified for mRNA expression were compared between infected kidneys from mouse different *DEFA1A3* copy numbers with no differences observed for (E) *Tlr2*, (F) *Tlr4*, and (G) *Tlr5*. (H) *Tlr6* kidney mRNA expression differences were significantly lower only for infected *DEFA*^*4/4*^ mice compared with littermates. **(I)** *Tlr9* kidney mRNA induction down-regulated for infected *DEFA*^*4/4*^ mice compared with infected littermates. No differences were recorded in vehicle-challenged mice. Data are represented as the mean CFU/ml or mRNA expression ± SD of saline or pyelonephritis multi-drug–resistant strain–challenged kidneys at 6 hpi. Differences were analyzed using one-way ANOVA and Tukey’s post hoc test for groups of mice ranging from 3 to 10 biological replicates.

To discern whether lower urinary tract bacterial burden drives reduced kidney Toll-like receptor signaling in a bacterial viability–dependent manner, we performed UPEC superinfection challenge with α-Defensin 1-3–resistant UPEC (MDR58) and analyzed inducible gene expression trends. After MDR58 challenges, kidney *DEFA*^*4/4*^ bacterial burdens between infected mice did not show differences compared with *DEFA*^*4/0*^ and *DEFA*^*0/0*^ mice (Fig 6A). Evaluation of Toll-like receptor downstream effector gene targets (*Il1β*, *Ifnβ*, and *Il6*) was further assessed to confirm acute protective immunomodulatory phenotypes in *DEFA*^*4/4*^ in the setting of MDR58 infections. Superinfection-challenged kidneys showed down-regulation of *Il1β* gene expression in a *DEFA1A3* copy number–dependent manner (Fig 6B). However, infected *DEFA*^*4/4*^ kidneys showed significant *Ifnβ* and non-significant *Il6* reduction in gene expression when compared to non-carrier mice (Fig 6C and D). We further confirmed anti-inflammatory effects are viability-dependent because of the lack of reduced *Tlr2* and *Tlr4* gene expression by comparing phenotypes with CFT073 and MDR58 superinfection challenges (Fig 6E and F). Similarly, a mild effect was also observed for induction of *Tlr5* gene expression (Fig 6G). After MDR58 challenge, *DEFA*^*4/4*^ kidneys show significantly reduced *Tlr6* expression and down-regulation of *Tlr9* transcript induction to baseline levels compared with littermate counterparts (Fig 6H and I). Furthermore, mice with *DEFA*^*4/4*^ copies exert a protective phenotype that is both bacterial viability-dependent and -independent of the transgenic *DEFA1A3* gene copies to decrease DAMP-associated inflammatory pathways. These results suggest α-Defensin 1-3 interactions with UPEC-associated Toll-like receptor ligands lead to differential Toll-like receptor signaling that results in a subsequent reduction in pro-inflammatory signaling to ameliorate both infection burden and inflammation in the kidney.

## Discussion

Although α-Defensin 1-3/*DEFA1A3* has been shown to be critical in UTI defense, the lack of a complete mechanistic understanding of its polymorphic role(s) during host innate immune defense response has impacted its translatability to impact care (48). AMPs, such as α-Defensin 1-3, have been primarily recognized to neutralize pathogens through the permeabilization of bacterial membranes, and interactions with virulence factors (49). Over the last decade, α-Defensin 1-3 (*DEFA1A3*) DNA CNVs have been associated with other infectious and autoimmune diseases (10, 14, 21, 28, 30, 50, 51, 52). These associations suggest gene dose–dependent indirect effects occur depending on the physiological context and warrant further investigation under appropriate infectious disease models such as UTIs (13, 14, 29, 30, 53, 54). In this study, we used a transgenic mouse carrying human *DEFA1A3* DNA copy numbers challenged with UPEC to demonstrate the gene dose–dependent mechanism of protection during acute UTIs. The host innate immune system responses comprise complex dynamics to mount an effective antimicrobial response while preventing exuberated inflammation and bystander collateral damage (55). Using the superinfection challenges, we demonstrate transgenic *DEFA1A3* copy numbers in mice drive a gene dose–dependent acute protection from UPEC; CFT073 invasion. Repeated challenges allowed for a more robust UPEC pyelonephritis model to identify UTI pathogenesis and innate immune responses in an immunocompetent non-refluxing murine background model (36).

During pyelonephritis, excessive PMNs transmigrate to infected kidney releasing granular effectors, including α-Defensin 1-3. Locally in the kidney, collecting duct epithelial cells react to bacteria and enact innate immune signaling responses (22, 37, 41, 56, 57). α-Defensin 1-3 have been characterized to be increased in culture-positive urine samples from UTI patients (18, 19). Prior studies have also positively correlated *DEFA1A3* DNA copy numbers and mRNA transcript levels in the kidney (10). However, the cellular source of α-Defensin 1-3 expression contributing to up-regulation across the urinary tract has not been dissected. After our superinfection model in transgenic *DEFA1A3* mice, we demonstrate myeloid cell depletion before challenge blunts α-Defensin 1-3 protective antimicrobial effects. The depletion of α-Defensin 1-3^+^Neutrophils suggests degranulation and release of translated peptides when transmigrated into the infected kidney site might be necessary to mount an antimicrobial host defense response against UPEC in conjunction with other neutrophil-specific factors. On the contrary, collecting duct intercalated cells induce and produce α-Defensin 1-3 at both transcriptional and translational levels upon infection. Studies using transgenic kidney transplant models have demonstrated that kidney *DEFA1A3* is the primary contributing source of defense against UPEC invasion (9). Results from neutrophil-depleted *DEFA*^*4/4*^ mice suggest one or more neutrophil-associated factor(s) might be needed to complement kidney-specific α-Defensin 1-3 antimicrobial effects. Thus, we can conclude IC sources contribute to the expression of α-Defensin 1-3 to drive a reduced urinary tract burden phenotype that is potentially dependent on neutrophil degranulation in the infected kidney.

The mammalian collecting ducts possess a diverse repertoire of AMPs that is spatially and temporally expressed during the orchestrated innate response against invasive uropathogens (25, 31, 58). Because of several potential α-Defensin 1-3–producing cellular sources during UTI, we show that the source of inducible *DEFA1A3* expression derives from ICs. Collecting duct ICs are characterized to perform distal acid–base homeostasis and production of AMPs such as α- and β-defensins, cathelicidin, lipocalin, calprotectin, and ribonucleases (11, 39, 40, 59). Non-ICs consist of all the other heterogeneous epithelial, endothelial, and interstitial cells that make up the kidney. Importantly, the enriched non-IC population might contain immature hybrid PC-IC cells, which can co-express Aquaporin-2 and low α-Defensin 1-3 levels (60). Hybrid PC-IC cells could account for mild mRNA increase in non-ICs and rare co-localization events from collecting duct luminal lining. The evidence further suggests that hybrid PC-IC cells can undergo high RNA velocity changes that potentially influence IC differentiation upon UPEC exposure (59). Altogether, the increasing α-Defensin 1-3 expression that protects the urinary tract is primarily sourced from the collecting duct intercalated cells in the kidney medulla. Collectively, other AMPs expressed at the kidney parenchyma and released into the medullary lumen could work in concert with collecting duct epithelial-derived *DEFA1A3* expression sources to protect from bacterial invasion.

AMPs have been recently shown to act in both direct and indirect mechanisms of bactericidal defense (23). Like cathelicidin-related peptides, α-Defensin 1-3 induce bacterial damage that subsequently modulates Toll-like receptor induction via interaction with ligands for bacterial cell wall components and unmethylated CpG-DNA complexes (61, 62). We demonstrate the interaction of potential Toll-like receptor ligands by gene dose–dependent *DEFA1A3* effects contributes to an anti-inflammatory phenotype through downstream reduction of IFN1β and IL-1β gene expression, and inflammatory macrophage recruitment. Lower serum pro-inflammatory cytokine responses have been reported in sepsis patients with >8 *DEFA1A3* CNs, suggesting an overlap between phenotypes observed in our murine study (27).

**Figure S6.**
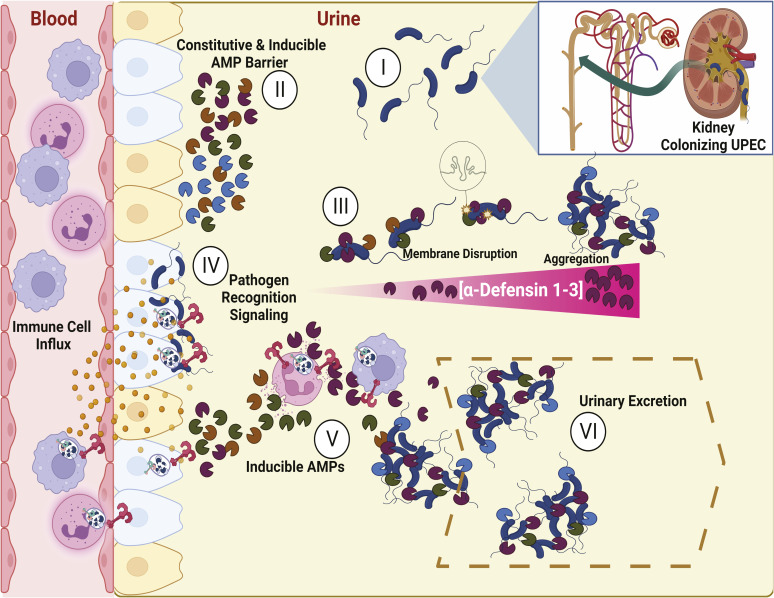
Proposed mechanism of kidney α-Defensin 1-3 gene dose–dependent protection against uropathogenic *E. coli* (UPEC) infection and inflammation. (I) Colonizing UPEC ascends ureters and infects the distal kidney collecting ducts. (II) Collecting duct epithelial cells produce a constitutive and inducible repertoire of antimicrobial peptides (AMPs) that form a barrier against invasive uropathogens. Variable levels of α-Defensin 1-3 during infections are expressed proportionally to the harbored host *DEFA1A3* gene copy number. (III) Alone and/or in concert with expressed AMPs, α-Defensin 1-3 induce membrane disruption and aggregation in a dose-dependent manner. (IV) Invasion of UPEC into the kidney leads to pro-inflammatory signaling and cellular influx via recognition of bacterial ligands through Toll-like receptors. (V) Induction of renal and extrarenal α-Defensin 1-3 from intercalated cells and neutrophils leads to agglutination of UPEC and associated components driving attenuated Toll-like receptor activities. (VI) Resulting interactions from the induction of α-Defensin 1-3 in concert with collecting duct–derived AMPs drive fine-tuning of pro-inflammatory cell infiltration, orchestrated bacterial damage, and subsequently excretion of agglutinated UPEC via urinary flow.

We report a differential Toll-like receptor response phenotype between murine low and high *DEFA1A3* copy-number mice. By interrogating the mRNA expression of Toll-like receptor superfamily and downstream effector cytokines, we captured bacterial viability–dependent and bacterial viability–independent modulation driven by murine *DEFA1A3* DNA copy numbers. Using α-Defensin 1-3–resistant UPEC, we were able to dissect the host defense mechanisms by which high DNA copy-number *DEFA1A3* and its expression enable modulation of Toll-like receptor pro-inflammatory signaling in a bacterial viability–dependent manner. These results further suggest Toll-like receptor signaling can be negatively regulated by increased α-Defensin 1-3 expression to prevent excessive inflammatory damage from pathogen-associated molecular patterns that are independent from direct antimicrobial effects against UPEC. Fig S6 summarizes the proposed direct and indirect mechanism of α-Defensin 1-3–dependent protection derived from our study findings. Initially, UPEC infects the kidney parenchyma and medulla (I). Medullary kidney regions are enriched with intercalated and principal collecting duct epithelial cells that release AMPs into the urinary lumen in both constitutive and inducible manners (II). AMPs are secreted at mucosal surfaces where bacteria contact DEFA–expressing cells and likely exist in a microenvironment with high local concentrations. The microenvironment results in dose-dependent UPEC membrane damage and agglutination antimicrobial effects before subsequent dilution of their concentration after release into the urinary lumen (III). After establishment of bacterial colonization of the kidney primary defenses, Toll-like receptors signal cytokines and chemokines for immune cell influx of PMNs (IV). Gene copy number–driven induction of α-Defensin 1-3 from intercalated cells in concert with neutrophil degranulation results in increased urinary AMP levels that drive enhanced membrane damage and agglutination effects against UPEC (V). Because of continuous urinary production in the kidney, the remaining agglutinated bacteria and pathogen-associated molecular patterns are removed through urinary flow (VI). The proposed combined mechanism of action indicates *DEFA1A3* gene dose–dependent expression protects the urinary tract by diminishing and neutralizing Toll-like receptor–associated bacterial ligands that lead to subsequent feedback pro-inflammatory signaling.

It is important to note our murine study has certain limitations. Surface and intracellular Toll-like receptor signaling underlies complex dynamics during the response to molecular signatures of bacterial infections, and studies with other pathogen recognition receptor families are warranted (63). Binding interactions between *TLR2* and *TLR6* heterodimers in other infectious settings have also been described (64). A recent report showed UPEC exposure to human ICs leads to significant up-regulation of TLR6 gene expression (65). TLR2 and TLR9 can have non-overlapping, opposing, and non-reductant effects in acute and chronic infectious disease settings (2, 66, 67). To further explore these diverse mechanisms at play, knockout and ligand-specific stimulations of candidate TLR targets would need to be applied to individually dissect novel *DEFA1A3*-mediated interactions under in vivo pyelonephritis models. Alternatively, Toll-like receptors (such as TLR4 and TLR5) can also have redundant functions that contribute to UTI susceptibility independently of evidenced antimicrobial gene dose-dependent effects nor immunomodulatory α-Defensin 1-3/*DEFA1A3* interactions (2, 4, 5, 6, 46). α-Defensin 1-3 potent agglutination on both live and damaged bacteria can be attributed to the ability to neutralize a diverse range of microbial toxins as previously eluted by others (68, 69). The AMPs tested in combination with α-Defensin 1-3 can elicit distinct antimicrobial effects when incubated at sub-killing concentrations against uropathogens. Cooperative interaction models of LL-37 and α-Defensin 1 have been elucidated, in which the AMPs induce distinct conformational membrane changes toward prokaryotic membranes (70). In our study, α-Defensin 1-3 peptides elicited agglutination of both live and damaged bacteria. Cathelicidin (LL-37) and α-Defensin 1-3 peptide mixtures with α-Defensin 1-3 enhanced agglutination effects exclusively for live bacteria. Ribonuclease 7 confers potent bacteriostatic activity but does not elicit agglutination. Cooperative effects using Ribonuclease 7 and α-Defensin 1-3 mixtures suggest augmentation of antimicrobial effects can occur independent of bacterial agglutination much like combinatorial effects of classic antimicrobial therapeutic agents. DMBT1 agglutinates but does not have bactericidal activity alone. In cooperative activity with α-Defensin 1-3, our data suggest that the large DMBT1 molecule (300–400 kD) allows for increased direct α-Defensin 1-3 antibacterial damage perhaps through anchoring microbes to the DMBT1 molecule. Our quantification approach could measure combinatorial effects between AMPs and α-Defensin 1-3 to promote bacterial agglutination, but we are limited to elucidate what additional mechanisms lead to bacterial killing. Future studies should dissect how AMP mixtures agglutinate uropathogens using techniques such as atomic force microscopy, which can shed insight into direct antimicrobial mechanisms attributed to conformational changes in bacterial cellular wall components and toxins. The enhancement of antimicrobial effects by AMP combinations can be evaluated at the physiological level using CRISPR approaches to study the role of compound genetic modifications in agglutinating AMPs. Our study sheds light on the combined direct and indirect *DEFA1A3* copy number–dependent protection of the kidney under normo-physiological innate immune response conditions. α-Defensin 1-3 induce agglutination at concentrations lower than its MIC, and its combinatorial effects in concert with other AMPs against pyelonephritis and pyelonephritis multi-drug–resistant strains demonstrate that AMPs are not required in high concentrations that may have deleterious effects such as host cytotoxicity or pathologic inflammatory cascade induction while being efficacious in killing microbes.

Future studies should elucidate whether copy number–dependent immune protective responses using the murine *DEFA1A3*-expressing cells are conserved in refluxing mice. Similar stratification findings from sepsis studies have characterized patients with more than 8 *DEFA1A3* copies to have a less robust pro-inflammatory response, which could be demonstrated by challenging transgenic *DEFA1A3* mice to urosepsis challenges (27). Therefore, experiments need to be performed with even higher copy numbers (>8) to expand our findings in the diverse human *DEFA1A3* CNV repertoire. In addition, the superinfection pyelonephritis murine models using other UTI-associated uropathogens that can also cause pyelonephritis such as *Proteus*, *Klebsiella*, *Enterobacter*, and *Staphylococcus* are further warranted. Potential findings from the applications of these models would confer an enhanced understanding of uropathogen immune invasion, multi-drug resistance, and pleiotropic α-Defensin 1-3 effects in other infectious and non-infectious diseases. Because of reported combination effects between AMPs, future studies that elucidate the cumulative contribution of other UTI disease risk genes in combination with *DEFA1A3* CNVs would expand the translatability of our findings. This can potentially offer a comprehensive stratification of disease recurrence and prophylaxis outcomes in susceptible vesicoureteral reflux or immunocompromised patients. Lastly, α-Defensin 1-3 host–pathogen molecular interactions in synergy with other urinary tract–associated AMPs expressed during UTIs can offer insight into future novel therapeutic modalities against bacterial-driven inflammation and drug-resistant uropathogens in an era of limited antibiotic efficacy.

## Materials and Methods

### Transgenic mouse generation

A mouse genetically engineered to express *DEFA1A3* was generated by Dr. Tomas Ganz when human α-Defensin 1-3/*DEFA1A3* gene copies were inserted into the murine genome using a bacterial artificial chromosome (BAC-10) construct as previously described (20). Our group characterized this mouse on a C57BL/6 background using long-range DNA sequencing and determined that each mouse possessed four copies of each gene per chromosome (9, 20, 21). To generate littermate mice with various *DEFA1A3* genome copies, WT C57BL/6J (Cat# 000664) mice were purchased from the Jackson Laboratory and bred in-house. Mouse breeding and experimental procedures were performed under approved IACUC protocol #20105 at the Laboratory Animal Resource Center at Indiana University School of Medicine.

### PCR of transgenic DEFA mouse genotyping

∼2 mm of mouse tail biopsy was subjected to DNA extraction and amplification with KAPA HotStart Mouse Genotyping Mix, and oligonucleotide sequences DEFA1A3_set 1 to generate a WT allele band forward primer 5′-TCTCACCTGAGGTTCCTGCT-3′ and reverse 5′-CCTGATGAGCTGATTGCAGA-3′; and DEFA1A3_set 2 used as forward 5′-GATGTTCACAGCAGGGGATT-3′ and reverse 5′-CCTGATGAGCTGATTGCAGA-3′ for the transgenic allele were combined and amplified using thermocycler conditions according to the manufacturer’s protocol (Cat# 2GFHSGKB; Roche). As previously demonstrated, 513- and 729-bp PCR products were analyzed in 2.5% electroporated agarose gel based on 1-kb DNA Hyper Ladder (Cat# N3232; New England Biolabs) (9).

### Murine pyelonephritis challenge experiments

*E. coli* pyelonephritis strain (UPEC; CFT073 or UPEC; MDR58) was grown overnight on Luria–Bertani (LB) media at 37°C in a orbital shaker at 5*g*. 8- to 16-wk-old anesthetized female C57BL/6J mice were infected with a 50-μl suspension containing 1 × 10^8^ CFU/ml of *E. coli* pyelonephritis strain in PBS or vehicle control as described (71). After 3 h of initial infection, transurethral infections were repeated for superinfection challenges (36). Prior studies have characterized the increased *DEFA1A3* mRNA expression and robust UTI burden after superinfection challenges (9). Mice were euthanized by CO_2_ inhalation at respective timepoints, and urinary tract organs were harvested and processed for consecutive experiments.

#### In vivo PMN depletion

Mice were injected via intraperitoneal route with 100 μg/mouse of monoclonal anti-mouse Ly6G/Ly6C (Gr-1) depletion antibody (Clone RB6-8C5, Cat# BE0075; Bio X Cell) or isotype IgG2b control (Cat# BE0090) 24 h before UTI challenges (72).

### Quantitative real-time PCR mRNA expression assays

Total RNA from organ or cell lysates was isolated and purified using RNeasy Plus Micro Kit. Following the manufacturer’s instructions, cDNA was prepared with the high-capacity cDNA reverse transcription kit (Applied Biosystems). The list of gene targets and catalog numbers used throughout the study can be found in Table S1. Singleplex qRT–PCR protocol was performed using Luna Universal qRT-PCR Master Mix (New England Biolabs). Target gene expression cycle thresholds were normalized to the housekeeping gene and calculated into relative expression using the 2^−ΔΔCq^ method (73).

Table S1 RT–qPCR reagent information used throughout the study. Predesigned probes and customized primer/probe sets for mRNA gene expression assays.

### Magnetic-activated cell sorting of kidney leukocytes, intercalated cells (ICs), and non-intercalated cells (non-ICs)

Kidneys from mice of different conditions and genotypes were aseptically collected in ice-cold 1x sterile PBS. Using gentleMACS dissociation (Miltenyi Biotec), single-cell suspensions were digested in Accumax enzymatic solution (Cat# AM105; Innovative Cell Technology) as previously described. Cells were filtered and supplemented with 1xDMEM before centrifugation for 10 min at 300*g*. Red blood cell lysis buffer (Cat# 420301; BioLegend) was applied to resuspend cells and incubated for 5 min on ice. After lysis, the cell suspension was supplemented with DMEM and subjected to filtering through a 70-μm nylon mesh (Cat# 22-363-548; Thermo Fisher Scientific). Cells were washed again and resuspended in EasySep buffer (Cat# 20144; Stem Cell Technologies) for subsequent annexin V separation using EasySep Dead Cell Removal Kit (Cat# 17899; Stem Cell Technologies). To isolate kidney-derived leukocytes, anti-mouse CD45 microbeads (Cat# 130-052-301; Miltenyi Biotec) were applied and incubated, and CD45^+^ cells were flushed out with 1 ml of magnetic-activated cell sorting buffer (PBS containing 0.5% BSA and 2 mM EDTA). The process was repeated using anti-mouse CD117 microbeads (Cat# 130-097-146) on CD45^−^ cell suspensions to flush out ICs (CD45^−^/CD117^+^). Non-ICs were recovered from CD117^−^ cells (9, 59).

### Enrichment of PMNs and flow cytometry studies

Whole-kidney single-cell suspensions were prepared as described above. Briefly, washed filtered cells were centrifuged without deceleration in 33% Percoll gradient (Cat# P1644; Millipore Sigma) for 20 min at 300*g* under 4°C. One million total PMNs were then subjected to the Fc binding blockade with anti-CD16/32 (Cat# 553142; BD Biosciences) for 15 min. After non-specific binding blocking, the mouse antibodies further described in Table S2 were incubated on ice for 30 min. Fixed viable cells were counted using Live/Dead cell dye and analyzed in the Attune NxT flow cytometer (Thermo Fisher Scientific). Supplemental gating strategy analysis of immune cells was applied as previously characterized (74, 75). The relative percent (%) change of immune cell populations in infected kidneys was calculated by dividing the mean counts from gated cell populations of two genotype groups and multiplication of the ratio by 100%.

Table S2 Flow cytometry antibody reagent information used throughout the study. Commercially available antibodies used for flow cytometry experiments to identify kidney-derived immune cell populations.

### Immunofluorescence microscopy

Tissues were dissected and fixed in 4% PFA overnight. After fixation, tissue blocks were prepared for histology in 70% ethanol. Slides were deparaffinized using Pro-Par Clearant (Cat# NC9537734; Anatech Ltd) three times in total before switching to a different container every 3 min. After rehydration and 1xPBS wash, slides were permeabilized at RT. Antigen retrieval was applied and washed again. Superblock (Cat# 37537; Thermo Fisher Scientific) was pipetted on the tissue for 5 min to block non-specific binding. Primary antibodies chicken anti-V-ATPase-E1 (Cat# GW22284F; Sigma-Aldrich), rabbit anti-V-ATPase-G3 (Cat# AB1220012; Abcam), and α-Defensin 1-3 (Cat# N5377-03P; US Biologicals) at 15 μg/ml were then added to the tissues and incubated at 4°C overnight. The next day, slides are washed off to remove residual antibodies. Secondary Alexa Fluor 488 anti-chicken, Alexa Fluor 488 anti-rabbit, and Cyanine3 anti-goat antibodies were respectively added and incubated in the dark for 30 min at RT at 1:600 dilution. After washing excess reagents, slides were mounted with Vectashield mounting solution with DAPI (Cat# H-1200-10; Vector Laboratories) and allowed to harden in the dark for at least 20 min before imaging in a Keyence BZ9000 microscope (Keyence Corporation).

### Organ bacterial colony-forming unit analysis

Mouse tissues were homogenized and serially diluted in a 96-well microplate containing sterile 1xPBS. Samples were added to LB agar plates and incubated statically at 37°C overnight for CFU quantification as previously demonstrated (9).

### Time/dose–response-kill UPEC assays

*E. coli* strains (K12; SMG123, UPEC; CFT073, and UPEC; MDR58) were cultured in LB media at 37°C with 5*g* orbital shaker conditions overnight. Mid-log bacterial suspensions (OD_600_ = 0.1) were further grown for the consecutive kinetic experiments. Sub-bacteriostatic concentrations of α-Defensin 1-3 (Cat# HC4014; Hycult Biotech) were added to each prepared 1–2 × 10^6^ CFU/ml *E. coli* inoculum in sterile 1xPBS (1, 10, 100 μg/ml). After OD_600_ reading at baseline, the cultures were further incubated and aliquoted for 1-, 3-, and 24-h post-inoculation readings in a round-bottom 96-well microplate (Cat# 3799; Corning). Control wells and testing conditions were performed in technical duplicates.

### Bacterial agglutination assays

*E. coli* strains under various conditions were visualized for viability under the microscope using Live/Dead BacLight Bacterial Viability Kit (Cat# L7012; Thermo Fisher Scientific). Human AMPs: cathelicidin peptide, LL-37 (Cat# AB140725; Abcam), Ribonuclease 7, RNase7, (Cat# AB164629), and recombinant Deleted in Malignant Brain Tumors 1, DMBT1 (kindly provided by Dr. Jan Mollenhauer), were added to media containing 1–2 × 10^6^ CFU/ml of mid-log bacterial suspensions, incubated, and imaged in a 96-well round-bottom microplate (Cat# 3799; Corning). An equal amount of SYTO9 and propidium iodide (PI) viability kit dyes was applied to each well separately and imaged under the EVOS microscope at a 20X objective fluorescent lens (Thermo Fisher Scientific). Quantification of surface area (μm^2^) for the largest 20 clumps was performed and compared across conditions tested in imaged wells using Celleste Imaging Image Software (Thermo Fisher Scientific).

### Figure design and statistical software applications

Data visualization in figures plotted was designed using GraphPad Prism 10 (GraphPad Software). Measured probability values at <0.05*, <0.01**, and <0.001*** represent significant difference symbols between groups were determined by a specified statistical test described in the figure legend. Supplemental scientific abstract Fig S6 was created with BioRender.com (license agreement #EW26LANXWC, Toronto, Canada).

## Supplementary Material

Reviewer comments

## Data Availability

Primary research data, statistical analyses applied, and methodologies are provided in the study results, figure legends, and uploaded supplemental materials. All raw data are available to share with the scientific community upon request.
