# Supplementary material for: DEFA1A3 DNA gene-dosage regulates the kidney innate immune response during upper urinary tract infection
Source: Life Sci Alliance. 2024 Apr 5;7(6):e202302462. doi: 10.26508/lsa.202302462 (PMC10997819; doi:10.26508/lsa.202302462)
Supplement: Supplementary file 1 [file LSA-2023-02462_TableS1.docx]

**Supplemental Table 1.**

| **Gene Target Symbol** | **Description** | **Catalog#** |
| --- | --- | --- |
| *Tlr2* | Toll-like receptor 2 | Mm00442346 |
| *Tlr4* | Toll-like receptor 4 | Mm00445273 |
| *Tlr5* | Toll-like receptor 5 | Mm00546288 |
| *Tlr6* | Toll-like receptor 6 | Mm00441868 |
| *Trl7* | Toll-like receptor 7 | Mm04933180 |
| *Trl9* | Toll-like receptor 9 | Mm00446193 |
| *Il1β* | Interleukin-1-beta | Mm00434228 |
| *Il6* | Interleukin-6 | Mm00446190 |
| *Ifnβ* | Interferon-1-beta | Mm00439552 |
| *Mpo* | Myeloperoxidase | Mm01298424 |
| *Gapdh* | Glyceraldehyde 3-phosphate dehydrogenase | Mm99999915 |
| *DEFA1A3 Forward Primer*  *DEFA1A3 Reverse Primer* | Human  α-Defensin 1-3 | FP: 5’-CCCTCGCCATCCTTGCTGCC-3’  RP: 5’- CTTGAGCCTGGATGCTTTGGAGCC-3’ |
