# Supplementary material for: DEFA1A3 DNA gene-dosage regulates the kidney innate immune response during upper urinary tract infection
Source: Life Sci Alliance. 2024 Apr 5;7(6):e202302462. doi: 10.26508/lsa.202302462 (PMC10997819; doi:10.26508/lsa.202302462)
Supplement: Supplementary file 2 [file LSA-2023-02462_TableS2.docx]

**Supplemental Table 2.**

| **Antibody Target** | **Clone** | **Fluorochrome** | **Catalog#** |
| --- | --- | --- | --- |
| Mouse CD45 | 30-F11 | Brilliant Violet 510 | BD563891 |
| Mouse Ly6G | 1A8 | FITC | BD561105 |
| Mouse Ly6C | AL-21 | PE-Cy^TM^7 | BD560593 |
| Mouse I-A/I-E | M5/114 | Brilliant Violet 421 | BD562564 |
| Mouse CD11c | HL3 | PE-CF594 | BD562454 |
| Mouse Singlec-F | E50-2440 | APC-Cy™7 | BD565527 |
| Mouse CD11b | M1/70 | Brilliant Violet 650 | BioLegend: 101239 |
| Mouse CD64 | X54-5/7.1 | Brilliant Violet 605 | BioLegend: 139323 |
| Mouse CD24 | M1/69 | PerCP/Cyanine5.5 | BioLegend: 101823 |
